# Neuronal Excitation Induces Tau Protein Dephosphorylation via Protein Phosphatase 1 Activation to Promote Its Binding with Stable Microtubules

**DOI:** 10.3390/neurolint16030049

**Published:** 2024-06-11

**Authors:** Sosuke Yagishita, Megumi Shibata, Akiko Furuno, Shuji Wakatsuki, Toshiyuki Araki

**Affiliations:** Department of Peripheral Nervous System Research, National Institute of Neuroscience, National Center of Neurology and Psychiatry, 4-1-1 Ogawa-Higashi, Tokyo 187-8502, Japan

**Keywords:** tau protein, phosphorylation, depolarization, microtubules, phosphatase

## Abstract

The tau protein is a microtubule-associated protein that promotes microtubule stabilization. The phosphorylation of the tau protein has been linked to its dissociation from microtubules. Here, we examined the relationship between neuronal depolarization activity and tau protein phosphorylation by employing model systems in culture as well as in vivo. The KCl-evoked depolarization of cultured neurons has often been used to investigate the effects of neuronal activity. We found dephosphorylation at AT8 sites (S202, T205), T212, AT180 sites (T231, S235), and S396 in KCl-simulated cultured neurons. We also found that the KCl-induced tau protein dephosphorylation increases the level of the tau protein fractionated with stable microtubules. In an in vivo experiment, we demonstrated that the exposure of mice to a new environment activates protein phosphatase 1 in the mouse hippocampus and induces tau protein dephosphorylation. We also found an increased amount of the tau protein in a stable microtubule fraction, suggesting that the dephosphorylation of the tau protein may lead to its increased microtubule association in vivo. These results suggest that the association of microtubules with tau proteins may be regulated by the tau protein phosphorylation status affected by neuronal electrical activity.

## 1. Introduction

The tau protein is a microtubule-associated protein that promotes microtubule stabilization [1,2]. Hyperphosphorylated and abnormally phosphorylated tau proteins form neurofibrillary tangles, observed in the brains of aged individuals and patients with Alzheimer’s disease, causing neuronal dysfunction [3]. Previous studies using in vitro models with purified tubulin and tau protein [4,5] or CHO cells expressing the tau protein [6,7] have shown that the binding of the tau protein with microtubules is reduced by tau protein phosphorylation. Thus, the phosphorylation of the tau protein has been associated with functional modifications in the physiology and pathology of the nervous system.

In studies using cultured neurons, KCl-induced depolarization is often used as a means of generating electrical excitation to mimic neuronal physiological activity [8,9,10,11,12,13,14,15]. Previous studies have shown that the depolarization of cultured neurons induced by KCl leads to tau protein dephosphorylation [16,17]. Previous reports have shown that phosphorylation at Ser214, Thr231, Ser262, and Ser356 reduces tau protein interactions with microtubules [6,18,19,20,21,22]. However, it is unknown whether KCl-induced dephosphorylation affects its association with microtubules. It is also unknown whether neuronal activity alters tau protein phosphorylation status. A recent study showed that dentate gyrus granule cells in the mouse hippocampus show a task-dependent small and transient depolarization of their membrane potential when an animal encounters a novel environment [23], suggesting that the cognition of a novel event causes neural depolarization. Here, we aimed to analyze the tau protein phosphorylation status and resultant events as a consequence of neuronal depolarization using models both in vitro as well as in vivo.

## 2. Materials and Methods

### 2.1. Animals

All animals were maintained in accordance with the guidelines of the National Center for Neurology and Psychiatry. The technical protocols for the animal experiments in this study were approved by the Committee on Ethical Issues in Animal Experiments of the National Center of Neurology and Psychiatry (approval number: 2023005, approval date: 8 February 2023). C57BL/6J mice and ICR mice were purchased from CLEA Japan (Tokyo, Japan).

For novel environment exposure experiments, C57BL/6J male mice (10–13 weeks old, 25–30 g, *n* = 51) were transferred to a novel environment consisting of a new cage, new bedding, and several plastic conical tubes (15 mL, 50 mL) for an indicated length of time (5 min–24 h) with free access to food and water and exposure to standard light–dark cycle (12 h–12 h).

### 2.2. Antibodies

For immunoblot analyses described in this work, we used the following primary antibodies: anti-acetylated tubulin (clone: 6-11B-1, ab24610, abcam, Cambridge, UK, mouse monoclonal, 0.1 μg/mL), anti-GAPDH (016-25523, Fujifilm, Tokyo, Japan, mouse monoclonal, 0.1 μg/mL), anti-phosphorylated tau protein (clone: AT8, #90206, Fujirebio, Gent, Belgium, mouse monoclonal, 0.2 μg/mL), anti-phosphorylated tau protein (Thr212) (44-740G, Thermo Fisher Scientific, Waltham, MA, USA, rabbit polyclonal), anti-phosphorylated tau protein (clone: AT270, #90207, Fujirebio, Gent, Belgium, mouse monoclonal, 0.2 μg/mL), anti-phosphorylated tau protein (clone: AT180, #MN1040, Thermo Fisher Scientific, USA, mouse monoclonal, 0.2 μg/mL), anti-phosphorylated tau protein (Ser396) (44-752G, Thermo Fisher Scientific, USA, rabbit polyclonal), anti-phosphorylated tau protein (Ser404) (clone: D2Z4G, #20194, Cell Signaling Technology, Danvers, MA, USA, rabbit monoclonal, 0.2 μg/mL), anti-Tau protein (clone: RTM38, Fujifilm, rat monoclonal, 0.5 μg/mL), and anti-Tubulin (clone: DM1A, T-9026, Sigma, St. Louis, MO, USA, mouse monoclonal, 0.1 μg/mL). We used the following secondary antibodies: anti-rabbit IgG HRP-linked antibody (#7074, Cell Signaling Technology, 1:5000), anti-mouse IgG HRP-linked antibody (#7076, Cell Signaling Technology, 1:5000), anti-rat IgG HRP-linked antibody (#7077, Cell Signaling Technology, 1:5000), and anti-goat IgG HRP-linked antibody (#705—35-003, Jackson Immunoresearch, West Grove, PA, USA, 1:5000).

### 2.3. Primary Cortical Neuronal Culture

Cerebral cortices were removed from day 15 embryonic mouse pups. Cells were dissociated with papain and seeded at a density of 5 × 10^5^ cells/well onto 24-well plates coated with poly-L-lysine (Merck, Darmstadt, Germany) and laminin (Merck) in DMEM containing 10% FBS. From the second day in vitro, the cells were maintained in Neuro-medium (Miltenyi Biotec, Bergisch Gladbach, Germany) containing 2% Neuro-Brew-21 (Miltenyi Biotec) and 1 mM GlutaMAX (Thermo Fisher Scientific). The cells were cultured for 14 d and used for analyses. Upon the harvest of cells, culture plates were placed on ice prior to harvest to minimize the effects caused by unstable temperature during manipulation [24]. For the examination of phosphatase inhibitors, tautomycetin (for PP1, with lower affinity for PP2A) (#2305, Tocris Bioscience, Abingdon, UK) [25] and okadaic acid (for PP1 and PP2A) (#495604, Sigma, St. Louis, MO, USA) were added to the culture at a final concentration of 1 μM and 50 nM, respectively, immediately followed by KCl stimulation.

### 2.4. Biochemical Analysis of Hippocampus

The isolated hippocampus was homogenized with Tris-buffered saline (TBS) containing a protease inhibitor cocktail (#25955-11, Nacalai tesque, Kyoto, Japan) and a phosphatase inhibitor cocktail (#07575-51, Nacalai tesque), and subject to ultracentrifugation at 125,000× *g*, 4 °C, 20 min to obtain supernatant for immunoblot analysis.

### 2.5. Microtubule Fractionation

Microtubule fractionation was performed according to a previous report [26]. Briefly, harvested primary cultured neurons or isolated hippocampi were immediately homogenized in an ice-cold microtubule-stabilizing buffer [MSB: 0.1 M MES, pH 6.8; 10% glycerol; 1 mM MgSO_4_; 1 mM EGTA; 0.1 mM DTT; 0.5% Triton X-100; 10 μM Taxol; 2 μM GTP] containing a protease inhibitor cocktail (#25955-11, Nacalai tesque, Kyoto, Japan) and a phosphatase inhibitor cocktail (#07575-51, Nacalai tesque). After centrifugation at 2400× *g* for 5 min to remove the debris, the supernatants were centrifuged at 100,000× *g* using a TLA-55 rotor (Beckman Coulter, Inc., Brea, CA, USA) for 20 min to obtain pellet as the microtubules with microtubule-associated proteins (P2 fraction). The resultant supernatants (S2 fraction) were further centrifuged at 500,000× *g* using a TLA 100.3 rotor (Beckman) for 60 min to separate the insoluble protein complexes in the precipitation (P3 fraction) from soluble proteins in the supernatant (S3 fraction). All fractions were dissolved in the SDS-sample buffer and boiled for 5 min prior to immunoblot analysis. 

### 2.6. Immunoblot Analyses

Proteins were separated on conventional 10% acrylamide gels, followed by transfer to PVDF membranes. The membranes were incubated with the indicated primary antibody overnight at 4 °C in TBS containing 0.05% Tween 20. After treatment with the horseradish peroxidase-conjugated secondary antibody for 30 min at room temperature, the immunoblot signal was visualized using ImmunoStar Zeta or ImmunoStar LD reagents (Wako, Osaka, Japan) and captured with the LAS-4000 (Fujifilm, Tokyo, Japan) or FUSION (M&S Instruments, Osaka, Japan) for analysis using Image J software(1.53a).

### 2.7. Phosphatase Assay

The phosphatase assay was performed according to a previous report [27]. Hippocampal tissues were isolated from mice in their home cage and mice after exposure to the novel environment. Isolated tissues were homogenized with TBS containing a protease inhibitor cocktail (#25955-11, Nacalai tesque) and subject to ultracentrifugation at 125,000× *g*, 4 °C, 20 min. Aliquots of the resultant supernatants were incubated at 37 °C for the indicated time with/without phosphatase inhibitors, followed by termination of the reaction with the addition of the SDS-sample buffer. The protein phosphatase (PP) inhibitors used in this study were tautomycetin (for PP1, with lower affinity for PP2A) (#2305, Tocris Bioscience, Abingdon, UK) [27], fostriecin (for PP2A) (#344280, Sigma), cyclosporin A (for calcineurin) (#239835, Sigma), and sanguinarine chloride (for PP2C) (#2302, R&D Systems, Mineapolis, MN, USA).

### 2.8. Statistical Analyses

Statistical analyses were performed using Prism 8 (GraphPad Software Inc., La Jolla, CA, USA). Results were expressed as the mean ± SEM. The significance of differences (* *p* < 0.05) between groups was examined using Student’s *t*-test and a one-way ANOVA followed by Dunnett’s multiple comparison test, or a one-way ANOVA followed by Sidak’s multiple comparison test, Dunnett’s multiple comparison test, or Tukey’s multiple comparison test.

## 3. Results

### 3.1. KCl Treatment Led to Tau Protein Dephosphorylation in Primary Cortical Neurons

**Figure 1:**
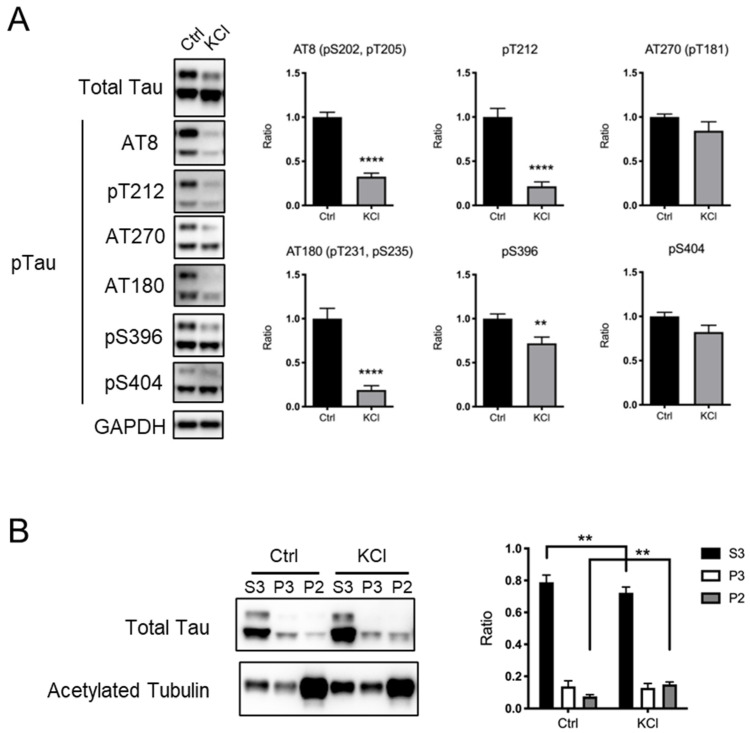
KCl-induced neuronal depolarization in culture causes tau protein dephosphorylation, leading to its increased capacity to bind to microtubules. (**A**) Representative immunoblots (**left**) and the quantified expression levels normalized to the control (**right**) showing expression of phosphorylated tau protein. Statistical analysis was performed using Student’s *t*-test. The graph shows mean ± S.E.M. (Ctrl, *n* = 11; KCl, *n* = 10). (**B**) Results of microtubule fractionation. Representative immunoblots (**left**) and the quantified ratio in each fraction were shown (**right**). Statistical analysis was performed using two-way ANOVA followed by Sidak’s multiple comparison test. The graph shows mean ± S.E.M. (Ctrl, *n* = 5; KCl, *n* = 5). Ctrl, control; KCl, KCl treatment; Tau, tau protein; pTau, phosphorylated tau protein; ** indicates *p* < 0.01, **** indicates *p* < 0.0001.

KCl-induced depolarization has often been used as a model to investigate the effect of neuronal activity in culture [8,9,10,11,12,13,14,15]. To examine whether the phosphorylation status of the tau protein is affected by KCl-induced depolarization, we generated a mouse hippocampal neuron primary culture and performed KCl stimulation. As a KCl-induced subcellular Ca^2+^ level increase was reported to last for up to 4 h [16], we harvested neurons 4 h after the addition of 50 mM KCl to the culture and examined the levels of tau protein phosphorylation at several sites, including the AT270 site (T181), AT8 sites (S202, T205), T212, AT180 sites (T231, S235), S396, and S404. We found that phosphorylation at AT8 sites, T212, AT180 sites, and S396 was significantly decreased by KCl treatment, while phosphorylation at the AT270 site and S404 was unchanged (Figure 1A). The levels of total tau protein were comparable between the Ctrl and KCl-treated neurons (Figure 1A). Previous reports suggested that phosphorylated tau protein is dissociated from microtubules [4,5,6,7,18,20,22]. To investigate whether KCl-induced tau protein dephosphorylation affects its association with microtubules, we performed microtubule fractionation using primary cortical neurons [25]. We found that KCl treatment increased the tau protein in stable microtubule fractions (P2, indicated by the presence of acetylated tubulin) and decreased the tau protein in free tubulin fractions (S3) (Figure 1B). These results suggest that KCl treatment causes the dephosphorylation of tau proteins at some key residues to increase its binding to stable microtubules.

### 3.2. Exposure to Novel Environment Decreases the Level of Phosphorylated Tau Protein

**Figure 2:**
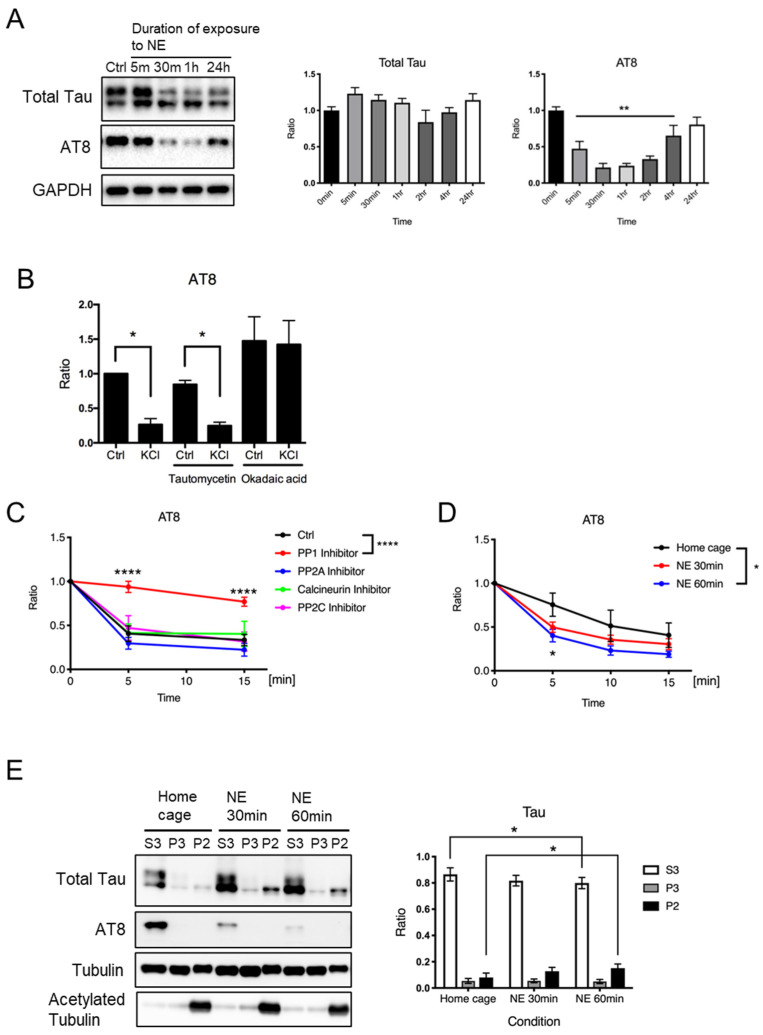
Exposure to a novel environment causes neuronal tau protein dephosphorylation, leading to its binding to microtubules in vivo. (**A**) Representative immunoblots (**left**) and the quantified expression levels normalized to the control (**right**) showing expression levels of tau protein and phosphorylated tau protein at AT8 recognition sites in hippocampal tissues obtained from wild-type mouse brains at indicated time after novel environment exposure. Statistical analysis was performed using one-way ANOVA followed by Dunnett’s multiple comparison test. The values show mean ± S.E.M. (*n* = 6~11). (**B**) A bar graph showing phosphorylated tau expression levels after KCl-induced neuronal depolarization in the presence of indicated phosphatase inhibitors in cultured neurons detected by AT8 antibody using immunoblot analysis. Expression levels are shown relative to the level of unstimulated control. The values are shown as mean ± S.E.M. (* *p* < 0.05; *n* = 3). Statistical analysis was performed using one-way ANOVA followed by Sidak’s multiple comparisons test. (**C**,**D**) Phosphatase assay results. In (**B**), quantified levels of AT8 immunoreactivity were measured by immunoblot analysis in lysates from untreated wild-type mouse brain incubated at 37 °C for indicated time with 1 μM of inhibitors of each phosphatase, i.e., PP1 (tautomycetin), PP2A (fostriecin), Calcineurin (cyclosporin A), or PP2C (sanguinarine chloride), relative to the level at time zero, are shown. In (**C**), quantified levels of AT8 immunoreactivity were measured by immunoblot analysis in lysates from brains of wild-type mice exposed to a novel environment for 30 min or 60 min, incubated at 37 °C for the indicated time, relative to the level at time zero, are shown. The values are shown as mean ± S.E.M. (*n* = 3–7). Statistical analysis was performed using two-way ANOVA followed by Dunnet’s multiple comparison test. (**E**) Mice were exposed to a novel environment for the indicated time, and their hippocampi were subjected to microtubule fractionation. Representative immunoblots (**left**) and the quantified levels of AT8 immunoreactivity shown as ratios in each fraction (**right**) are shown. Statistical analysis was performed using two-way ANOVA followed by Tukey’s multiple comparison test. The values show mean ± S.E.M. (*n* = 6). Tau, tau protein; Ctrl, control; NE, new environment; * indicates *p* < 0.05, ** indicates *p* < 0.01, **** indicates *p* < 0.0001.

To investigate whether neuronal activity leads to tau protein dephosphorylation in vivo, we examined tau protein phosphorylation in the mouse cerebral cortex after exposure to a novel environment for various periods of time (from 5 min to 24 h). The AT8 sites, which are often hyperphosphorylated in brains with Alzheimer’s disease [28,29,30,31,32,33], are among the KCl-sensitive phosphorylation sites in this study (Figure 1) and the previous studies [16,17]. Therefore, we examined AT8 site phosphorylation status after novel environment exposure in mouse hippocampus. We found that the levels of phosphorylated tau protein at the AT8 sites were significantly reduced after new environment exposure for 5 min to 4 h (Figure 2A). The levels of total tau protein were comparable up to 24 h after exposure to a novel environment. These results suggest that neuronal depolarization caused by novel environment exposure may lead to tau protein dephosphorylation in vivo.

Previous reports showed that increased tau protein phosphorylation in physiological settings is caused by decreased phosphatase activity [26,34]. Based on these observations, we speculated that the dephosphorylation of tau proteins after neural activity might be caused by increased phosphatase activity. To examine this possibility, we added tautomycetin or okadaic acid (inhibitors for PP1/PP2A) to the neuronal culture and performed KCl stimulation. We found that the addition of the inhibitors may affect the AT8 site phosphorylation (Figure 2B), but we do not think we can conclude anything from this experiment because the inhibitors could also affect the activity of kinases that phosphorylate tau proteins, or modify signaling that mediates KCl-induced neuronal depolarization. To examine the role of phosphatase activity in regulating the phosphorylation status of tau proteins in response to neuronal activity more directly, we performed a phosphatase assay using brain tissues obtained from mice with or without novel environment exposure. Brain lysates were incubated at 37 °C with 1 μM of inhibitors for each phosphatase, i.e., protein PP1 (tautomycetin), PP2A (fostriecin), calcineurin (cyclosporin A), and PP2C (sanguinarine chloride), followed by the quantification of the level of AT8-positive tau protein. We confirmed a decrease in the level of AT8-positive tau protein in the absence of any inhibitor (Figure 2C, Ctrl). We found that the decline was significantly suppressed by the PP1 inhibitor tautomycetin, but not by any other inhibitors (Figure 2C). These results demonstrated that PP1 might be a potential phosphatase that dephosphorylates the AT8 sites. To investigate whether exposure to a novel environment induces an increase in phosphatase activity, we prepared brain lysates from mice in a home cage or mice after exposure to a novel environment for 30 min or 60 min and performed the phosphatase assay to evaluate PP1 activity by examining AT8 phosphorylation. We found that the lysates derived from mice exposed to a novel environment for 60 min showed significantly higher PP1 activity compared to lysates derived from mice in a home cage (Figure 2D). These results suggest that exposure to a novel environment may activate PP1.

### 3.3. Exposure to a Novel Environment Increases the Levels of Tau Protein Fractionated with Stable Microtubules

To examine whether novel environment exposure-induced tau protein dephosphorylation affects its function, we analyzed the association of tau proteins with microtubules using a microtubule fractionation assay using hippocampal tissues. We found that exposure to a novel environment for 60 min resulted in an increase in the level of tau protein fractionated into a stable microtubule fraction (P2) and a decrease in the level of tau protein fractionated into free tubulin fraction (S3) (Figure 2E). These results suggest that tau protein dephosphorylation caused by exposure to a novel environment may promote its binding to stable microtubules.

## 4. Discussion

Previous reports have shown that tau protein phosphorylation is elevated during different physiological processes, including development, hibernation, hypothermia, sleep, intermittent hypoxia, and brain ischemia [35,36,37,38,39,40,41,42,43,44,45]. However, the relationship between tau protein phosphorylation and neuronal activity is still unknown. To the best of our knowledge, our study is the first to report that exposure to a novel environment, a physiological stimulus to induce neuronal activity, leads to tau protein dephosphorylation and an increase in the level of tau proteins in stable microtubule fractions in vivo. We also found that PP1 activity is induced by novel environment exposure in mice. These results suggest that tau protein phosphorylation and its association with microtubules is regulated by neuronal electrical activity. 

Similar to our current observations, previous reports have also demonstrated KCl treatment-dependent tau protein dephosphorylation [16,17], but the residues showing KCl treatment dependency in phosphorylation status seem to vary in different reports. Here, we found that the AT270 site (T181) and S404 are KCl-insensitive phosphorylation sites, while others reported that they are dephosphorylated by KCl treatment [17]. Since different experimental conditions were used in the reports, these data may suggest that the activity of kinases/phosphatases could be differentially regulated by varying degrees of depolarization. It is also possible that the substrate specificity of each kinase/phosphatase is not tightly determined and/or is affected by circumstantial conditions and post-translational modifications other than phosphorylation. Since the substrate specificity of PP1 is known to be affected by a number of different conditions, including interaction of other molecules and structural modifications [46,47,48], the PP1-dependent dephosphorylation status of tau proteins might also be affected by different experimental conditions.

We show here that neuronal depolarization leads to tau protein dephosphorylation, followed by an increase in its binding with microtubules in vitro and in vivo. We have no sufficient data here to extend the discussion on our current finding to human disease, so we do not think it is appropriate to talk about the putative influence of neuronal activity on tau protein aggregation at this point. While the results suggest the existence of mechanisms regulating tau protein phosphorylation in physiological conditions, further study is required to clarify whether the currently identified mechanism could be extended to that for pathological phosphorylation that causes aggregate formation in neurodegenerative disorders, including Alzheimer’s disease and post-ischemic brain neurodegeneration [45]. 

## Data Availability

All the data are shown in the paper.
